# Co‐Production, Co‐Creation and Co‐Design in Primary Health Care: A Scoping Review of Design, Implementation, Impact and Sustainability

**DOI:** 10.1111/hex.70447

**Published:** 2025-10-04

**Authors:** Cate Dingelstad, Nicolette Hodyl, Rebecca Wyse, Suzanne Lewis, Alison Koschel, Nicholas Goodwin

**Affiliations:** ^1^ University of Newcastle, Hunter New England Central Coast Primary Health Network, Hunter Medical Research Institute Newcastle New South Wales Australia; ^2^ Hunter Medical Research Institute, Hunter New England Local Health District Newcastle New South Wales Australia; ^3^ University of Newcastle Newcastle New South Wales Australia; ^4^ Hunter New England Central Coast Primary Health Network Tamworth New South Wales Australia; ^5^ Centre for Research in Health System Performance, Yong Loo Lin School of Medicine, National University of Singapore Singapore

**Keywords:** co‐creation, codesign, coproduction, health systems, impact, participatory design, primary health care, sustainability

## Abstract

**Introduction:**

The terms coproduction, co‐creation and codesign (called the ‘Three‐Cs’ in this article) are applied to activities that include stakeholders in the design and implementation of health services. This scoping review sought to understand how Three‐Cs approaches have been designed and implemented sustainably in the primary health care context.

**Methods:**

Three databases (Medline, Embase and CINAHL) were searched for articles published between 2013 and 2024 using key words related to the Three‐Cs and primary health care (Appendix A) and limited to articles available in English. Dual blind review against specific inclusion and exclusion criteria was carried out at both title/abstract and full text screening stages. The SPICE Framework was used to consider design, implementation, impact and sustainability. An assessment of quality and risk of bias was completed using the Joanna Briggs Institute (JBI) Critical Appraisal Checklist [1]. The Framework to Assess the Impact of Translational (FAIT) health research [2] was used to review and assess the impact of the design and implementation of Three‐Cs approaches in each study.

**Results:**

Thirty articles were included in this review with 33% (*n* = 10) providing a clear definition of their Three‐Cs approach: 10% (*n *= 3) identified their approach as Coproduction, 20% (*n* = 6) as co‐creation, and 70% (*n* = 21) as Codesign. Implementation contexts included clinical settings (10%, *n* = 3), for example prediabetes programs; community settings (50%, *n* = 15) such as care navigation support; and health systems settings (40%, *n* = 12) including health service development. Approaches were implemented across metropolitan (37%, *n* = 11), regional (43%, *n* = 13), and rural (17%, *n* = 5) settings.

Three‐Cs approaches in primary health care settings were most typically used for health promotion, and to support improved health equity and health access. Key outcomes included novel solutions to problems, improvements to health and health systems, and solutions that met consumer needs. While nine of the 30 studies undertook some form of evaluation, limited evidence on impact and sustainability was found (7%, *n* = 2), where only two studies assessed whether the change that was implemented using a Three‐Cs approach was maintained in the longer term.

**Conclusion:**

Three‐Cs approaches have been adopted across a variety of primary health care settings, yet evidence for impact and sustainability is currently limited. More research is needed to evaluate how the Three‐Cs may best be implemented to support the long‐term sustainability of programs designed by such approaches. Future work should focus on developing a primary health care specific framework or guidance for implementing and evaluating Three‐Cs models, particularly in low‐resource settings and in typically underrepresented populations. This study will extend to consider how the FAIT assessment process can help to support the development of much needed implementation guidance.

## 1 Introduction

The World Health Organization (WHO) defines primary health care (PHC) as a “whole of society approach to effectively organize and strengthen national health systems to bring services for health and wellbeing closer to communities” [1]. At a systemic level, strengthening PHC has for many years been shown to lead to improved population health, increased access to care for all people, and improved quality and more cost‐effective healthcare provision. For these reasons, one of the World Health Organization's priority recommendations is to orient health systems towards PHC as the foundation of universal health coverage [2].

PHC is premised on the ability of all people to have both the right and access to the highest attainable level of health. This can occur through the coordination of health services that meet the needs of people throughout their lives, and which address the broader determinants of health through multi‐sectoral policy and action. In particular, the WHO specifically recognises the need for “health‐conducive environments in which individuals and communities are empowered and engaged” in maintaining and enhancing their health and well‐being [3], so providing for an approach that is centred on people's needs and preferences [4].

Yet PHC systems worldwide are under‐resourced [5], do not usually take a ‘whole‐of‐system’ perspective, and have become increasingly fragmented [6]. Fragmentation (lack of integration of health care delivery) occurs when delivery is distributed among numerous providers who do not coordinate effectively [7]. This can adversely impact the quality of care received by populations, creating inequitable access to care, and leading to increasing health care costs [8]. Given this context, an evidence‐based and sustainable foundation for a coordinated approach in PHC is urgently needed.

A key approach to coordinating and strengthening PHC has been to build the skills and resources of individuals and communities as empowered users of health and care services. There is good evidence for the positive impact of a range of strategies and tools to support this including supported self‐management for health conditions, shared decision‐making, peer support, and support from family and caregivers [9].

At a systems level, approaches to engaging and empowering communities through ‘identifying and unlocking’ community assets have also demonstrated success in reducing inequalities [10], improving quality of life, and supporting community resilience, but only as long as there is ‘authentic Coproduction’ with communities during the process [11]. However, evidence suggests that the rhetoric supporting people‐centred care is not being matched by the reality of delivery where fragmented and passive involvement of people and communities persists [12]. Such evidence suggests that the reinvigoration of collaborative approaches will be important if the challenges faced by fragmented PHC systems are to be addressed.

Collaborative approaches with people in communities in health systems has focused on the key concepts of Coproduction, co‐creation and Codesign (the ‘Three‐Cs’). Co‐creation is focused on collaboration and creative problem solving between diverse stakeholders at all stages of a piece of work from problem identification and solution generation through to implementation and evaluation [13, 14]. Codesign is the active collaboration between stakeholders to design solutions to a set problem [13, 14]. Coproduction includes the two active elements of co‐creation and Codesign and encompasses all co‐approaches. It is an activity or process where all key stakeholders work together to create something new, for example, knowledge, services, technologies [13, 14].

There are already several frameworks available to support the implementation of the Three‐Cs, including but not limited to the Double Diamond Framework (Design Council UK, 2005), the Patient Engagement Framework (Carmen, 2013), and the Codesign Framework (Sanders and Stappers, 2014). However, most previously developed frameworks are focused on the secondary and tertiary health care sectors, and few are specific to the PHC context. This could suggest a degree of tokenism towards effective engagement of people within PHC and a persistent power imbalance that favours systems and professionals as opposed to people and communities [15]. Given the effectiveness of PHC is based on a premise of fully engaged people and communities, understanding how the Three‐Cs can best be implemented within the PHC setting, including to support sustainable new PHC practices, represents a critical missing piece of knowledge.

This scoping review has two core aims: to understand how the Three‐Cs approaches have been designed and implemented in the PHC setting; and to assess the impact and sustainability on PHC when Three‐C approaches have been used. These aims help to address the knowledge gap around what has previously been done, whether these approaches have been impactful, and how they have been sustained. When this knowledge gap has been filled, primary care practitioners and policymakers will be in a better position to understand how best to develop guidance to implement acceptable and appropriate Three‐Cs interventions. Such interventions are then more likely to be sustained long term and will give greater opportunity for improvements in health service delivery and health outcomes in PHC settings.

Key terms within this review are defined as follows: *design* refers to the development of an intervention, activity, or initiative; *implementation* refers to the delivery of an intervention, including how it has been modified to suit local need; *impact i*s the demonstration of value to the community and to the system; and *sustainability* is the continued capacity, continued delivery and continued receipt of benefit from the intervention, activity, or initiative [16]. To our knowledge, this is the first scoping review to systematically evaluate how the Three‐Cs have been designed, implemented, and sustained in primary health care settings globally, using both the JBI [17] and FAIT [18] Frameworks.

## 2 Methods

The SPICE Framework (Table 1) was used to structure the search strategy and the concepts of design, implementation, impact and sustainability were addressed. The inclusion and exclusion criteria used to define the search is outlined in Table 2.

**Table 1:** SPICE framework.

**S**etting	Primary health care settings in Australia and internationally
**P**opulation	Community members, primary health care providers (clinicians), primary health care system administrators.
**I**ntervention	Primary health care delivery using Three‐Cs approaches.
**C**omparison	Measures used. For example, pre post.
**E**valuation	Improved health and wellbeing outcomes for the community, improved outcomes for the PHC system, sustained outcomes over time.

**Table 2:** Inclusion and exclusion criteria.

Inclusion criteria	Exclusion criteria
Any type of service or approach within a primary health care context. Person‐centred care including health literacy, shared decision making, shared care assessments, supported self‐care. Any PHC service approach using Coproduction, co‐creation and/or Codesign. Has information on developed tools. For example, frameworks, guidelines, procedures etc.	Not in PHC setting, for example in a hospital or specialist setting. Does not meet the definition of co‐creation, Codesign or Coproduction. Does not provide information on methods or approach to using the Three‐Cs. Protocols, conference abstracts, studies without an abstract except where the title specifically relates to the Three‐Cs. The study only undertakes a survey or questionnaire of end users in the methodology. The study only includes one group of stakeholders (e.g., clinicians) as part of the Three‐Cs activity, and does not include multiple groups of stakeholders (e.g., community members or patients and carers). The study only focuses on research rather than implementation, service innovation or service redesign. The study only focuses on the development of curricula or educational incentives for service providers. Studies or commentaries looking at perceptions and issues of engagement with/in community health councils and committees. Studies examining patient participation in care/patient‐professional relationships/use of decision aids/health coaching. Approaches for patient or community activation and impact of these.

The review is reported according to the Preferred Reporting Items for Systematic Reviews and Meta‐Analyses (PRISMA) 2020 Statement [19] (Appendix C). Three databases (Medline, Embase and CINAHL) were searched using key terms listed at Appendix A, and limits applied around publication date (2013 to 2024), peer‐reviewed journals, and English language. The review team chose these three databases based on where literature relating to primary health care and participatory action research is most commonly published. A 10‐year time frame was applied due to this being a relatively new area of research, and the search was limited to publications available in English. Citation review of the 30 included articles was undertaken. Grey literature was searched using search engines, by reviewing websites of relevant key organizations that support or advocate for Three‐Cs approaches, and by reviewing authored reports on the Three‐Cs with similar inclusion criteria (*n* = 50, Appendix D). A critical appraisal was undertaken on each of the grey literature articles using the same inclusion and exclusion criteria to determine their relevance.

To address the two key aims, the following search terms were used: patients, carers, consumers, healthcare professionals, community, Coproduction, co‐creation, Codesign, lived experience, consumer consultation, patient public involvement, citizen science, health 4.0, participatory action research, public participation and involvement, metropolitan, regional, rural, remote, general practice, primary health care. Literature searches were conducted in June 2023 and updated in March 2024.

Retrieved references were uploaded into EndNote 20 bibliographic software (Clarivate Analytics, PA, USA) then exported to the Covidence review management platform (Veritas Health Innovation, Melbourne, Australia. Available at www.covidence.org) and duplicates removed. Each title and abstract, and then each full text article, were reviewed by two reviewers, conflicts discussed, and a consensus decision made on inclusion. Relevant data were extracted from each full text article by one reviewer and recorded in a purpose built excel spreadsheet. Data extracted included publication year, setting (metropolitan, regional, rural), study design, focus of the work (e.g., clinical, community, health systems), types of stakeholders involved, nature of the Three‐Cs being used (e.g., Coproduction, co‐creation or Codesign), components of the Three‐Cs, tools used, main findings, and the recommendations made. For all identified articles, an assessment of quality and risk of bias was completed using the JBI critical appraisal checklist [17, 20] for qualitative research. One person reviewed each article against the 10 questions in the JBI checklist, and this assessment was reviewed by the other authors.

To understand impact, each of the 30 articles was submitted to an assessment using the Framework to Assess the Impact of Translational (FAIT) health research [17]. The FAIT assessment can be applied prospectively or retrospectively and has been applied in this study in a retrospective way [21]. This approach enables understanding of the flow of logic in how impact has been achieved through the design and implementation of each Three‐Cs approach. From that assessment, three focus areas were identified and a summary of the impacts was determined.

## 3 Results

**Figure 1:**
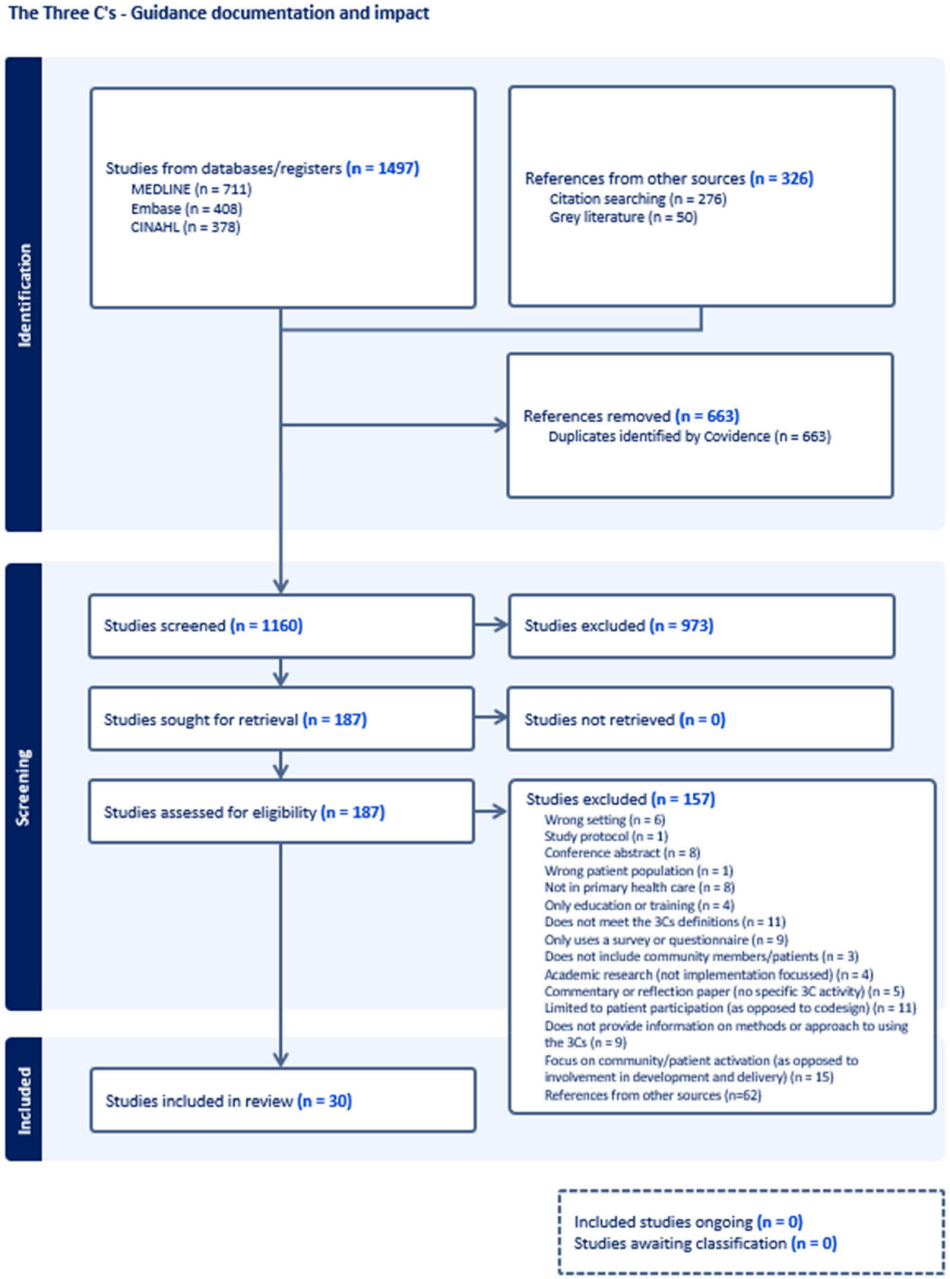
PRISMA diagram.

The literature search (Appendix A) identified 1497 articles from the databases and 326 references from other sources (Figure 1—PRISMA diagram). Of these, 1160 articles were screened. Twenty‐one full‐text articles were identified that addressed aim one and 9 full‐text articles were identified that addressed aim two (Appendix B). The JBI assessment reviewed each of the 30 articles for bias and quality. Over 85% of the articles scored a yes against each of the 10 questions using the inclusion criteria (Table 2) for the research. Of the small number of articles that scored a ‘no’ against one of the ten questions (*n* = 4), these concerns were discussed amongst all authors and a decision made on inclusion. This means that all 30 articles were considered to have little to no bias and the quality of the articles was good. It is noted that some of the questions within the JBI checklist were not relevant; for example, quality improvement projects are system improvement and therefore are exempt from ethics processes. Following a critical appraisal process, it was found that the grey literature (Appendix D) did not include any new or additional topics outside of what was found in the main review.

### 3.1 Study Design

By incorporating the qualitative narratives extracted from this review, the FAIT Framework was used to capture the complex stories of how the outcome and impact of the Three Cs emerged. This supported an understanding of the practical way in which the Three Cs approach was designed and implemented in their different contexts. This approach, therefore, was adopted to potentially allow for a more comprehensive understanding of how impact was achieved.

Essentially, the FAIT Framework supports analysis that examines (1) the aim of the study; (2) the planned activities undertaken to support its design and implementation; (3) the outputs from this, specifically who the participants were and what they did; and (4) the impact that was observed. The FAIT Framework structure is incorporated into Table 3 that provides an assessment of each of the articles included in the review.

**Table 3:** Assessment of included studies using the FAIT Framework.

Study #	Author, year, country	Stated health or health service need	Aim of study	Design *(setting and location)*	Implementation *(activities)*	Participants	Outputs and impact	Sustainability	Evidence‐based translational OR Discovery research
Studies with a health promotion focus									
1	Careyva, B.A. (2020) USA	Increasing pre‐diabetes prevalence.	*Develop a shared decision aid. *Evaluate tool feasibility.	Clinical setting, regional location	*Model for Improvement used to cocreate tool. *Implementation evaluation.	Patients, primary care clinicians, endocrinologists, pharmacy, diabetes educators, community members.	*Development of a pre‐diabetes decision aid was successful. *Clinician consult time was not extended.	Behaviour change was assessed using a survey six weeks following implementation and demonstrated success within this timeframe.	Evidence‐based translational research
4	Coteur, K. (2022) Belgium	Increasing sleep disorders.	*Develop evidence‐based educational material for patients.	Health systems setting, regional location	*Codesign workshops.	Primary health care professionals (HCPs), patients.	*Improved patient health literacy and self‐management regarding sleep disorders through development of a patient leaflet.	The six months post implementation evaluation demonstrated positive use in practice. Sustainability was supported through two additional collaborations.	Discovery
5	Cowdell, F. (2020) UK	Improving atopic eczema self‐management strategies for patients and primary health care consultations for patients and practitioners	*Devise strategies to amend patients and practitioner eczema mindlines.	Community setting, regional location	*Co‐creation workshops.	Patients, HCPs.	*Five key messages were identified for use in patient practitioner interactions. *Simple, consistent, evidence‐based knowledge is shared across patient–practitioner social boundaries promoting shared understanding.	Sustainability considerations were mentioned in relation to building on previous work. No evaluation was reported in this study.	Discovery
9	Grindell, C. (2022) UK	Understanding knowledge mobilisation.	*Understanding how Coproduction, Codesign and co‐creation approaches achieve knowledge mobilisation in the management of health conditions, *Understanding the extent to which knowledge mobilisation is accomplished.	Health systems location	*Systematic review.	HPCs, policy makers, organisations.	*People were brought together as active and equal partners. *All types of knowledge were valued. *Creative and iterative prototyping approaches to understanding and solving problems were used. *Co‐approaches show promise in achieving successful knowledge mobilisation to improve the way health conditions are managed.	This review also noted the lack of evaluation within the 24 included studies. The authors noted that sustainability and long‐term impact can be achieved through local champions driving the implementation.	Evidence‐based translational research
11	Holmqvist, M. (2023) Sweden	Incorporating medication plans into a patient's electronic health record.	*Creating a medication plan prototype to support older persons and HPCs jointly in using and monitoring medications.	Clinical setting, regional location	*Remote (web‐based) Codesign workshops. *Descriptive statistics review. *Interviews and questionnaires.	Patients, HPCs.	*A medication plan prototype was developed. *Remote Codesign approaches require careful planning. *Remote Codesign is inclusive of participant perspectives and facilitates learning by sharing experiences.	Evaluation was proposed for the next stage of implementation for this study. Sustainability considerations were not mentioned.	Discovery
12	Hunter, B. (2020) Australia	Providing effective systematic quality improvement (QI) functionality to improve chronic disease management.	*Co‐designing an electronic chronic disease quality improvement tool incorporating audit and clinical decision support for use in general practice.	Health systems setting, metropolitan location	*Codesign workshops. *Online Codesign and think aloud sessions.	General practice staff, patients.	*A dashboard for chronic kidney disease management was developed. *QI systems designed with end users that provide actionable options are most likely to succeed and be sustained over time. *Algorithms developed using data from electronic medical records (EMRs) can accurately identify patients at risk of chronic health conditions in primary care and support QI through audit and feedback.	The authors noted that end user input impacts positively on sustainability. Further piloting and evaluation were proposed for the next stage of implementation for this study.	Evidence‐based translational research
14	Mabetha, D. (2023) South Africa	Supporting health equity through community participation.	*Conducting theory‐informed analysis of community power‐building in primary health care (PHC). *Developing practical guidance to support participation as a sustainable PHC component.	Health systems setting, rural location	*Introduction workshops. *Engage and observe workshops.	Patients, HPCs, health services.	*A practice framework was developed to expand community power in PHC. *Three power perspectives were identified. *Agreement was gained to re‐establish a CHW support structure that had previously existed with clinic outreach teams. *The Participatory Action Research process was successful and planned to be embedded into clinic processes. *Community training was planned.	The authors noted that community power was successfully sustained during implementation. No evaluation was undertaken post implementation.	Evidence‐based translational research
15	Mahmood, S.S. (2020) Bangladesh	Engaging communities in local‐level planning and management of healthcare delivery.	*Instilling social accountability into monitoring mechanisms of health facilities. *Implementing community scorecards (CSC) in rural Bangladesh.	Health systems setting, rural location	*Codesign workshops. *Consensus workshops.	Patients, HPCs, health services.	*CSC raises awareness about health in the community and facilitates structured monitoring of services. *Positive changes were achieved including quality and accountability in health service delivery, community participation in health, revenue generation for health, and raising community awareness.	The implementation was found to be feasible and acceptable; however, sustainability was noted to be dependent on ongoing support and resourcing. No evaluation was undertaken post implementation.	Discovery
17	Morris, R.L. (2021) UK	Involving patients and carers in patient safety and prevention.	*Developing a patient safety guide for primary care (PSG‐PC).	Health systems setting, metropolitan location	*Codesign workshops. *Prototype development.	Patients, HPCs.	*Three key themes (the importance of communication, understanding roles and responsibilities, and developing partnerships) were identified to inform the development of the PSG‐PC. *The PSG‐PC can address national and international patient safety priorities. *The PSG‐PC involves patients in their patient safety by enabling patients to take responsibility of their care in partnership with HPCs.	No evaluation or evidence of sustainability were covered in this study.	Discovery
18	Neal, P. (2018) Haiti/USA	Ascertaining the health, education and economic needs of the Haitian population.	*Understanding of the comprehensive health needs of a rural Haitian village. *Empowering the community to be effective agents of change.	Health systems setting, rural location	*SWOT analysis in collaboration with community leaders. *Community involvement in implementing change.	Community members and leaders.	*Community engagement and empowerment is the key to promote sustainable community health initiatives.	Sustainability was a key focus. The study authors planned to rerun the tools developed 12 months post implementation to determine long term effectiveness and sustainability. No other evaluation was reported in this study.	Discovery
20	Ouellet, B. (2022) Canada	Children from families with low income are at an increased risk of developmental delays.	*Producing a tool adapted for families with low income that informs parents about early child development.	Community setting, metropolitan location	*Codesign workshops.	Low‐income families, parents of children aged 0‐5 years.	*A life‐sized grow chart was developed with an attached booklet giving expected outcomes for children and support numbers. *The ‘Technique of Research of Information by Animation of a Group of Experts’ (TRIAGE) method was adaptable and effective in mobilising a heterogeneous group of participants for the purpose of selecting priority information needs for the tool and choosing the most appropriate format to reach families with low income.	Evaluation planned but not yet progressed. Sustainability was not specifically covered.	Evidence‐based translational research
24	Sellen, K. (2022) Canada	Evidence‐informed overdose first aid kits should be developed with those with lived experience.	*Developing an overdose education and naloxone distribution (OEND) programme.	Community setting, metropolitan location	*Codesign workshops. *Advisory council workshops.	Community members, those with lived experience, pharmacies, family medicine clinics, addictions medicine clinics, emergency departments.	*A contextually appropriate solution was designed enabling anyone to be a lay responder to overdose.	Evaluation planned for subsequent phases of the project. Sustainability was not specifically covered.	Discovery
25	Silcock, J. (2023) UK	In older people living with frailty, polypharmacy can lead to preventable harm like adverse drug reactions and hospitalisation.	*Increasing the engagement of older people living with frailty in the process of deprescribing.	Community setting, regional location	*Introductory meetings. *Codesign workshops.	Older people, carers, HPCs.	*The deprescribing pathway developed provides a person‐centred, clinician‐facilitated approach to deprescribing consultations in primary care.	Evaluation planned but not yet progressed. Sustainability was not specifically covered.	Discovery
26	Sundram, F. (2017) New Zealand	Adolescents with mild‐to‐moderate depression could be supported with computerised cognitive behavioural therapy (cCBT).	*Developing an electronic monitoring (e‐monitoring) tool that monitors mood, risk, and treatment adherence of adolescents.	Health systems setting, regional location	*Focus groups.	Adolescents with depression, HPCs.	*An e‐monitoring web‐based cCBT tool was designed in line with clinician need for oversight of adolescents using Smart, Positive, Active, Realistic, X‐factor (SPARX) thoughts. The e‐monitor is also a system that is easy to use with information and alerts.	No evaluation was possible due to resourcing constraints. Sustainability considerations were not covered in this study.	Discovery
27	Timothy, A. (2021) South Africa	Optimal immunisation program service delivery and childhood vaccine coverage is lacking in South Africa.	*Improve immunisation service delivery in children under 24 months in an identified community within South Africa.	Community setting, metropolitan location	*Weekly radio sessions. *Nurse‐led education sessions. *Focus groups.	Community members, HPCs and providers.	*Co‐designed, local‐level interventions resulted in improvements in parents'/guardians' knowledge about immunisation, parent engagement and service provider commitment to improvement in immunisation service quality. *Community health literacy was improved.	Implementation occurred for 4‐6 months, then evaluation was undertaken. The authors noted that sustainability and long‐term impact can be achieved through local champion/s driving the implementation.	Evidence‐based translational research
28	Valaitis, R. (2014) Canada	Meeting the needs of our ageing population requires a more responsive approach.	*Using a persona‐scenario exercise to Codesign the health service intervention TAPESTRY (Teams Advancing Patient Experiences: Strengthening Quality).	Community setting, regional location	*Codesign workshops. *Persona development. *Scenario development.	Patients, volunteers, HPCs and providers.	*Early engagement with key stakeholders helped foster support and uptake of the pilot program. *Participants introduced many novel ideas that had not been previously identified.	No evaluation or evidence of sustainability were covered in this study.	Discovery
29	Valaitis, R. (2019) Canada	An increase in chronic conditions for older adults leads to complexity in management and treatment, resulting in challenges in coordinating care and communication across multiple providers.	*Using a modified approach to the persona‐scenario method to Codesign a complex primary health care intervention (Health TAPESTRY) by and for older adults and providers.	Community setting, metropolitan location	*Codesign workshops. *Persona development. *Scenario development.	Patients, volunteers, HPCs and providers.	*The ideas generated were incorporated into the Health TAPESTRY program's design and implementation. *Persona‐scenario workshops are effective in engaging stakeholders.	Participatory design was used with evaluation processes built into implementation in the short term. Sustainability was not specifically covered in this study.	Discovery
**Studies with a health equity focus**									
2	Cheng, C. (2020) Australia	Increasing digital literacy to improve health and equity outcomes.	*Codesign an eHealth solution using the Ophelia (Optimising Health Literacy and Access) process.	Community setting, metropolitan and regional locations	*eHealth literacy questionnaire (needs assessment tool). *Semi‐structured interviews. *Codesign workshops.	Users of primary health care services.	*A suite of vignettes supporting digital health literacy and an eHealth literacy questionnaire were developed *Stakeholders were effectively engaged through use of Codesign processes.	No follow up evident post the study demonstrating how the Ophelia process assisted solution development in the digital context.	Evidence‐based translational research
3	Cheverton, J. et al. (2016) Australia	Improving systems through value co‐creation approaches.	*Facilitate collaboration, coordination and integration between service providers.	Community setting, metropolitan location	*Codesign workshops. *Collaborative completion of a funding submission.	Service providers, users of mental health care services.	*Improved navigation and coordination between service providers. *Improved user experience.	Evaluation undertaken post implementation; some positive assumptions regarding impact are reported.	Evidence‐based translational research
7	Ekawati, F.M. (2023) Indonesia	Providing adequate maternity care in low‐and‐middle‐income countries during pandemic disruption.	*Codesign a maternity model of care.	Health systems setting, regional location	*Codesign workshops.	HCPs, patients, obstetricians.	*Key inclusions were health screening, breastfeeding care, limiting visitors, using telemedicine, and providing multidisciplinary team care in the new model of care.	No evaluation or evidence of sustainability were covered in this study. The study indicated that the next step was to pilot the new model of care.	Discovery
8	Foley, K.A. (2019) USA	Providing women's health screening for medical, behavioural, nutritional, psychological and social determinants of health.	*Create a comprehensive women's health screening tool incorporating intimate partner violence.	Community setting, regional location	*Codesign workshops.	HCPs, patients.	The women's health screening tool is a first step towards addressing screening barriers from both primary care providers' and community women's perspectives.	No evaluation or evidence of sustainability were covered in this study.	Evidence‐based translational research
10	Hertel, E. (2019) USA	Achieving truly patient‐centred health care.	*Assessing the process and impact of a co‐designed clinic service with equal numbers of patient and clinical provider/staff participants.	Health systems setting, regional location	*A 4‐day design event including structured observation. *Participant interviews and surveys. *Check in event post implementation.	Patients, clinical staff, organisations.	*The evaluation outcomes supported the greater and more effective use of partnerships with patients in care design.	Evaluation report demonstrating the success of using equal numbers of patients and staff in design events. This evaluation was conducted 15 months post implementation.	Evidence‐based translational research
**Studies with a health access focus**									
6	Davie, S. (2019) Canada	Providing easy access to afterhours primary health care services.	*Understand and address barriers to accessing afterhours services. *Increase community awareness of afterhours services.	Clinical setting, metropolitan location	*Model for Improvement used to Codesign approaches to access. *Patient interviews. *Codesign workshop to develop print materials for marketing.	Primary health care organisation, patients, community members.	*A poster with tear off sheet advertising afterhours services was developed and email communications established. *Increased community awareness of and ease of access to afterhours services was achieved.	Measures were established and evaluated every six months post implementation, up until 3 years post implementation demonstrating ongoing improvements.	Evidence‐based translational research
13	Knowles, S. (2018) UK	Effectively managing complex patients living with multimorbidity.	*Co‐designing novel interventions to address safety issues for patients with multimorbidity in primary care. *Assessing the approaches to translate research findings into intervention suggestions.	Health systems setting, metropolitan location **s**	*Codesign workshops. *Trigger film development. *Online survey.	Patients with multimorbidity in general practice, primary health care practitioners.	*Participants valued the opportunity to jointly propose new interventions and agreed to focus on polypharmacy. *The interaction between patients’ capacity, the demands of their treatment and the opportunities for intervention in primary care is emphasised.	An evaluation survey was open for two months following the final workshop receiving limited input with ambivalent responses from participants on the implementation.	Evidence‐based translational research
16	Mistry, S.K. (2022) Australia	Providing navigation‐related support to culturally and linguistically diverse populations in Australia.	*Evaluating the Codesign of an intervention in general practice settings to help address navigation problems.	Community setting, metropolitan location	*Pre‐workshop consultations. *Codesign workshops.	Culturally and linguistically diverse populations in Australia, general practices, service providers, primary health care practitioners.	*Bilingual Community Navigators (BCN) could help to address the problems faced by the culturally and linguistically diverse patients in accessing care in the Australian general practice setting.	No evaluation or evidence of sustainability were covered in this study. The study indicated that the next step was to evaluate the BCN model.	Discovery
19	Nimegeer, A. (2016) Scotland	Involving local stakeholders in reconfiguring rural service provision relevant to the region.	*Assessing the customised participation process implemented for rural community health services design.	Community setting, rural location	*Codesign workshops. *Interviews.	Community members, health services.	*Health services should be clear about the parameters and constrain possible options to those that can be supported to fruition. *Community health literacy and health system literacy is improved.	An evaluation was planned for four years post implementation. The authors noted that end user input impacts positively on sustainability.	Discovery
21	Reddy, A. (2019) USA	Over 1.1 million older adults (65 + ) are at risk of a major adverse drug event resulting from misuse of over the counter (OTC) medications.	*Developing a blueprint for participatory design in a community pharmacy context.	Community setting, regional location	*Codesign workshops.	Older adults, community pharmacy.	*Pharmacy staff can view older adults searching for OTCs and alleviates the time constraint on OTC counselling. *Prescription information accessibility is improved, aiding pharmacists in recommending safe OTC products to older adults.	The participatory design method used includes an initial evaluation and a formal evaluation. The authors noted that end user input impacts positively on sustainability.	Evidence‐based translational research
22	Roach, P. (2023) Canada	Access to health care for Indigenous peoples who live in rural, remote, northern and geographically isolated Canadian communities.	*Monitoring and measuring virtual and/or remote‐based primary care using an Indigenous patient experience tool.	Community setting, regional location	*Phone interviews. *Advisory group.	Indigenous community members, service providers.	*The Access, Relationships, Quality and Safety (ARQS) tool offers a way for Indigenous patients to provide feedback and for clinics to measure the quality of virtual primary care for Indigenous patients. *Access and quality of virtual primary care delivery for Indigenous patients may be advanced.	No evaluation or evidence of sustainability were covered in this study. The study indicated that the next step was to evaluate the ARQS tool.	Evidence‐based translational research
23	Robinson, A. (2022) UK	Inequalities negatively impact ethnic minority populations when accessing medicines advice.	*generating recommendations to improve access to medicines advice from community pharmacies for people from ethnic minority communities.	Community setting, regional location	*Codesign workshops. *Online workshops and interviews.	Community members, community pharmacies.	*A platform was provided to share the voices of members from ethnic minority communities and generate person‐centred recommendations to improve access to seeking medicines advice from community pharmacies. *Support is provided to community pharmacy services to overcome ethnic inequalities affecting medicines advice	No evaluation or evidence of sustainability were covered in this study.	Discovery
30	Yadav, U.N. (2021) Nepal	People with chronic obstructive pulmonary disease (COPD) in Nepal are not receiving adequate support to self‐manage their chronic condition.	*Co‐designing a model of care to support self‐management for people with multi‐morbid COPD in rural Nepal.	Health systems setting, rural location **s**	*Sharing workshops. *Government consultation. *Codesign workshops. *Advisory group development.	Patients, carers, HPCs and providers, policy makers.	*The Codesign approach was effective in engaging various stakeholders and in developing a model of care for rural Nepal. *There was value in using both top‐down and bottom‐up approaches to develop the new COPD model of care.	Evaluation is noted as important but not undertaken for the model of care developed. The authors noted that resourcing is a key sustainability enabler.	Discovery

The 30 articles included in this review were conducted in the following countries: United Kingdom (*n* = 7), Canada (*n* = 6), United States (*n* = 5), Australia (*n* = 4), South Africa (*n* = 2), and Bangladesh, Belgium, Indonesia, Nepal, New Zealand and Sweden (*n* = 1 each). The article authors reported their Three‐Cs approach as follows: Coproduction 10% (*n* = 3), co‐creation 20% (*n* = 6), and Codesign 70% (*n* = 21). Only 10 of the 30 articles (33%) provided definitions of what their use of the Three‐Cs term encompassed. The data collection methods used in the 30 articles included: surveys, teleconferencing, observation, one‐to‐one discussion, small group workshops, focus groups, facilitator and participant notes, documentation analysis, and case study analysis.

The Three‐Cs approaches were applied in a variety of PHC settings, including 50% (*n* = 15) in community settings (examples include navigation support and tool development); 40% (*n* = 12) in health systems settings (examples include health service development and knowledge mobilisation); and 10% (*n* = 3) in clinical settings (examples include prediabetes and medication planning). With respect to location, 43% (*n* = 13) were conducted in a regional setting, 37% (*n* = 11) in a metropolitan setting, and 17% (*n* = 5) in a rural setting (note that one of the included studies was a systematic review (Study 9 (Grindell, 2022) Appendix B) and is therefore not represented in these numbers). Sixty‐six percent (*n* = 20) of the articles used qualitative approaches (using nonnumerical data to inform the conclusions), 17% (*n* = 5) used mixed methods (using both qualitative and quantitative data), and 17% (*n* = 5) were either quality improvement studies (a process to measure continuous improvement) or a systematic review.

### 3.2 Study Implementation

The type of research undertaken in the studies was classified into either evidence‐based translational research (i.e., building understanding on whether and how an approach could work in a scalable way in real world settings. 47%, *n* = 14) or discovery research (i.e., creating evidence for a tool or process to be developed. 53%, *n* = 16). The focus of each study was identified as either a health promotion activity, such as knowledge sharing (60%, *n* = 18), a health equity activity, such as care navigation and service coordination (17%, n = 5), or a health access activity, such as after‐hours access to PHC (23%, *n* = 7). Health promotion in this study is viewed as encouraging healthy behaviours and preventing diseases; health equity is about everyone receiving fair and just access to health resources; and health access is about everyone being able to obtain health care services.

The target populations of the 30 articles encompassed: end users of primary health care (100%, *n* = 30); ethnic minority populations including Australian Aboriginals, culturally and linguistically diverse communities in Australia, and Hispanic people in the USA (27%, *n* = 8, Studies 1, 3, 7, 15, 16, 18, 22, 23); people with specific health challenges like eczema, cardiovascular disease, chronic kidney disease, Type 2 Diabetes, and chronic obstructive pulmonary disease (23%, *n* = 7, Studies 1, 4, 5, 12, 13, 26, 30); rural communities (23%, *n* = 7, Studies 7, 14, 15, 18, 19, 22, 27); older adults (17%, *n* = 5, Studies 11, 21, 25, 28, 29); health professionals (13%, *n* = 4, Studies 6, 10, 24, 27); and women (2%, *n* = 2, Studies 8, 20).

To explore further the areas of research where and how the Three‐Cs had been applied, we recorded tools and frameworks either used or developed in the process (Table 4), as well as information about the challenges and limitations experienced using each approach (Table 5).

**Table 4:** Tools and frameworks cited.

Tools/Framework used	Summary of tool	Study #
Experience‐based codesign	A collaborative and inclusive method used in service design to enhance a program, service, or product. It uses the experiences of service users and providers to inform the design.	6, 13, 17, 27
Evidence‐based design methodology	Relies on research and data to inform design decisions.	6, 12
Root cause analysis	Enables understanding of the underlying cause of a problem and develops solutions to address it.	6
Participatory design and action research techniques	These approaches emphasise collaboration with stakeholders who are part of the community being affected or studied.	7, 18‐22, 24‐26, 28‐30
eHealth literacy questionnaire (Optimising Health Literacy and Access – OPHELIA)	Designed to improve health outcomes and equity by enhancing health literacy and maximising access to health information and services [22].	2
The model for improvement	Provides a framework for developing, testing and implementing changes to the way that things are done that will lead to improvement.	1, 6, 12
The consolidated criteria for reporting qualitative research (COREQ) checklist	Provides researchers with a framework to report important aspects of the research team, study methods, context of the study, findings, analysis and interpretations.	23
Value co‐creation	A collaborative process where businesses and consumers work together to create value.	3
Co:Create Coproduction Matrix	Eight principles form the basis of the Co:Create Coproduction Matrix. They include being holistic, resourced, transparent, inclusive, iterative, positive, equal and sustainable.	5
The design thinking model	The five stages of design thinking are: empathise, define, ideate, prototype, and test. This is an iterative process.	30
Codesign Framework	This framework supports three approaches to Codesign: probes, toolkits and prototypes.	4
Design Innovation Framework	This approach fosters innovation through human‐centered design. It focuses on understanding user needs, generating creative solutions, and iterating based on feedback.	8
Patient Engagement Framework	A multidimensional model designed to enhance patient and family engagement in healthcare. This framework outlines various forms and levels of engagement, aiming to improve healthcare quality, efficiency, and population health.	10
Double Diamond Framework	Used to guide innovation and problem‐solving and is divided into four key phases: discover, define, develop and deliver.	11, 16
Emancipatory Power Framework and Limiting Power Framework	These are analytical tools designed to understand and address power dynamics in a community empowerment initiative by enhancing health equity and social justice.	14
Theoretical Framework of Acceptability	Provides a framework to assess the acceptability of healthcare interventions from the perspectives of both those delivering and receiving the interventions.	15
The Ladder of Citizen Participation	Illustrates different levels of citizen involvement in decision‐making processes.	19

**Table 5:** Challenges/limitations to the achievement of impact and sustainability.

Overview of challenges/limitations	# of times mentioned across all 30 studies See Appendix B for study titles.
Allocating appropriate time (e.g., adequate time is required to effectively complete Coproduction, co‐creation, or Codesign processes).	*n* = 9 (3, 5, 7, 21, 25, 27, 28, 29, 30)
Trust and rapport development (e.g., this must be built over time with key stakeholders).	*n* = 5 (3, 8, 20, 21, 29)
Involving sufficient participants (e.g., people, clinical sites and/or encounters).	*n* = 7 (1, 7, 10, 21, 24, 26, 29)
Involving representative participants (e.g., they represent the cohort being assessed).	*n* = 6 (12, 17, 20, 26, 29, 30)
Involving engaged participants (e.g., there are clear parameters on recruitment requirements and the scope of the participant role).	*n* = 5 (4, 11, 13, 26, 29)
Cultural and/or language barriers (e.g., through consultation with relevant stakeholders, culturally safe practices are put in place).	*n* = 5 (4, 18, 22, 23, 30)
Adequate funding is available to achieve the planned outcomes (e.g., funding the development of an online tool).	*n* = 4 (4, 10, 15, 30)
Participant capabilities (e.g., to navigate online tools and processes).	*n* = 4 (4, 6, 16, 22)
Power dynamics (e.g., power and control should be equal)	*n *= 3 (5, 13, 14)
Group think considerations (e.g., a decision may be too quickly made driven by a desire to reach a consensus, requiring planned mitigation strategies).	*n* = 3 (15, 21, 29)
Low participation rates (e.g., stemming from a reduced sense of participant ownership of the project direction or outcomes).	*n* = 3 (2, 15, 27)
Knowing the unknown (e.g., participants may miss harms or risks not known to them).	*n* = 3 (17, 19, 28)
Change inertia (e.g., giving up long held processes to move to a systems perspective).	*n* = 1 (3)

### 3.3 Study Impact

The Framework to Assess the Impact of Translational (FAIT) health research [17] was used to understand the impact of the Three‐Cs design and implementation for each of the 30 articles.

A summary of the impacts discussed in the 30 articles included:

- Improved communication between patients and service providers (12/30 studies).
- Consumer needs are better met, including hearing and valuing minority groups (7/30 studies).
- Elevated consumer awareness, health literacy and health system literacy (4/30 studies).
- Diverse and/or novel solutions are generated (4/30 studies).
- Service providers are not disadvantaged during consultation time (1 study).
- Written information is complemented and supported using Three‐Cs activities (1 study).

The evaluation undertaken for each of the 30 studies was varied; 9/30 studies undertook some form of evaluation: Three studies (10%) were evaluation reports (Studies 2, 10, 27. Appendix B), one study (3%) had evaluation planned but had yet to progress (Study 20, Appendix B), and five studies (17%) undertook an evaluation either within 6 months or just post 6 months for the initial Three‐Cs project (Studies 1, 3, 4, 6, 13. Appendix B).

The benefits of using Three‐Cs approaches, particularly in regional and rural research and with underrepresented populations were evident in the studies (Table 3). Including the perspectives of the end users in the design of any program that aims to benefit them will influence its success and long‐term impact.

### 3.4 Study Sustainability

Only two studies (7%) reported long term sustainability outcomes: One study (Study 6, Appendix B) conducted a 3‐year follow‐up to assess sustainability. Another study conducted an evaluation 15‐months post implementation to determine its long‐term impact (Study 10, Appendix B). Both studies demonstrated sustainability through continued impact from the implementation over the times indicated.

Table 5 lists the challenges or limitations in using a Three‐Cs approach as noted by the authors of the 30 articles. These could have had an impact on sustainability.

## 4 Discussion

This review assessed 30 articles from the literature to understand how Three‐Cs methodologies have been designed and implemented in PHC settings, and to understand the impact and sustainability of using the Three‐Cs approach in this setting. The included articles illustrated the breadth of contexts and clinical areas of interest where Three‐Cs approaches can be applied, and the different frameworks and tools that can support their use.

The benefit of using Three‐Cs approaches can be seen from the impact achieved in all 30 included studies, both during and immediately following implementation, and in one case, the impact (improved access to PHC after‐hours) had been sustained for 3 years following implementation. Given that only five of the studies specifically examined whether outcomes were sustained over time, further research comparing the longer term follow up of programs or services designed using Three‐Cs methodologies to other approaches will be needed to identify those factors that facilitate or prevent sustainability.

Such work is necessary to assess likely critical challenges in applying the Three‐Cs approach, especially in sustaining relationships and power dynamics to overcome issues such as professional and institutional resistance and community fatigue that have often been neglected [23]. Conversely, given that the strength of Three‐Cs approaches is that they include multiple key stakeholders and end‐users in their design and implementation, it may be anticipated that the outcomes from such approaches are more relevant, appropriate, feasible and acceptable than those developed using other methodologies, and are therefore more likely to be sustained over time [24].

The challenges of implementing a Three‐Cs approach, as outlined in Table 5, indicate that the engagement and empowerment of people in the development of PHC services may not be sufficient to support ongoing sustainability, despite this being one of the core components of a successful PHC system [3, 4]. Indeed, evidence suggests that sustainable Coproduction is contingent on the nature of the service and the environment in which it operates [24]. Sustainability of the Three‐Cs, then, is likely to result from an interplay between how the approach is designed and structured, the resources and skills available, and the ongoing relationships that support people's commitment over time. This implies that policy makers and funders must consider the implications around the time required to enable a Three‐Cs approach. Adequate time and resourcing must be allocated to support the development of trust and rapport, while ensuring appropriate representation of those impacted by the area being studied. An additional key consideration involves ensuring that power is equitably distributed among all participants engaged in the Three‐Cs approach to increase the likelihood of achieving sustained impact.

As demonstrated by the 30 studies there is currently a gap in the evidence around the sustained impact of Three‐Cs approaches in PHC. This corroborates recent review findings that have demonstrated a fragmented approach to assessing the Three‐Cs that lack the adoption of comparative research designs which could address such gaps [25]. However, the review did highlight the generic components of Three‐Cs approaches that have been used in design and implementation processes, so emphasising findings from previous reviews that the process requires, amongst others, a well‐defined focus, the embedding of end user engagement, effective communication, and the building of trustful relationships [26].

The high number of frameworks and tools used in the studies included in this review demonstrates inconsistent approaches to applying the Three‐Cs in practice in the PHC setting. Indeed, most existing frameworks used are based on approaches that focus on secondary and tertiary care settings. The development of specific guidance or a roadmap to support the ever‐increasing application of the Three‐Cs in PHC settings is required. The evidence developed through this review provides a basis to develop a roadmap to inform future approaches to the design and implementation of Three‐Cs methods and the significant potential of using the FAIT Framework [17] to support guidance and as a methodology to understand both impact and sustainability.

### 4.1 Limitations

The findings of this scoping review should be considered in the context of the strengths and limitations of the approach. The terminology associated with the review topic posed challenges; the lack of an established definition for each of the key words used and the broad use of these terms across the literature meant that the number of articles retrieved by each search were significant and some articles may have been missed due to the search parameters. The definitions used within this study are the predominant definitions used across the literature.

The search was limited to articles written in English and published in the last 10 years from 2013 through to 2024, and was conducted across three databases, which may have excluded some relevant literature.

Participatory approaches are essential for the development of an effective and appropriate solution; however, participant diversity and cultural differences may limit the generalisability of Three‐Cs approaches. This question will be explored further in the future research associated with this scoping review.

## 5 Conclusion

Understanding how to improve the PHC system from a population, cost, equity, and outcomes perspective is high on the agenda of many agencies within health systems. This review provides an understanding of how Three‐Cs approaches can and have led to desirable impacts in areas such as health promotion, health equity, and health access. Yet the review also highlights that Three‐Cs methodologies require a planned approach with sufficient resourcing and time to effectively implement, evaluate, and sustain. At present, the evidence is insufficiently robust to understand how best to implement the Three‐Cs to support sustainable impact.

Further work is therefore required to test the efficacy of Three‐Cs approaches in the PHC context. Future work should focus on developing a PHC‐specific framework or guidance for implementing and evaluating Three‐Cs models, particularly in low‐resource settings and in typically underrepresented populations. Such guidance will be essential if the Three C's approach can overcome the observed lack of effective participatory processes in primary health care. This will enable consistency of approach and build understanding of what underpins a successful approach to Three‐Cs implementation. The FAIT assessment process is a useful analytical tool to understand design, implementation, impact, and sustainability of the Three‐Cs and can help to support the development of much needed implementation guidance.

## Author Contributions

**Cate Dingelstad:** conceptualisation (lead), writing – original draft (lead), formal analysis (lead), writing – review and editing (equal). **Nicolette Hodyl:** writing – review and editing (equal). **Rebecca Wyse:** writing – review and editing (equal). **Suzanne Lewis:** methodology (lead), writing – review and editing (equal). **Alison Koschel:** writing – review and editing (supporting). **Professor Nicholas Goodwin:** conceptualisation (supporting), writing – original draft (supporting), writing – review and editing (equal).

## Conflicts of Interest

The authors declare no conflicts of interest.

## Data Availability

The data that support the findings of this study are available in the supplementary material of this article. The data that support the findings of this study are also available from the corresponding author upon reasonable request.

## 1

**The Three Cs in Primary Care—Medline search strategy**

1. Community Participation/
2. co?design*.mp.
3. co?produc*.mp.
4. co?creat*.mp.
5. co?construct*.mp.
6. co?develop*.mp.
7. "consumer consult*".mp.
8. "patient public involvement".mp.
9. "Health 4.0".mp.
10. 1 or 2 or 3 or 4 or 5 or 6 or 7 or 8 or 9
11. Primary Health Care/
12. exp General Practice/
13. General Practitioners/
14. "general practice".mp.
15. "primary health?care".mp.
16. Community Health Nursing/
17. "allied health".mp.
18. "community pharmacy".mp.
19. "community‐based health".mp.
20. 11 or 12 or 13 or 14 or 15 or 16 or 17 or 18 or 19
21. 10 and 20
22. limit 21 to (english language and yr="2013 ‐Current")

## 1

Scoping review article reference list.

**Table:**

Study #	Title	Publication date	Lead author	Journal	DOI
**1**	Designing and Evaluating a Prediabetes Shared Decision Aid	2020	Beth A. Careyva, MD	Journal of the American Board of Family Medicine	10.3122/jabfm.2020.02.190070
**2**	Co‐designing eHealth and Equity Solutions: Application of the Ophelia (Optimising Health Literacy and Access) Process	2020	Christina Cheng	Frontiers in Public Health	10.3389/fpubh.2020.604401
**3**	The Partners in Recovery program: mental health commissioning using value co‐creation	2016	Jeff Cheverton & Tina Janamian	Medical Journal of Australia 204 (7) j 18 April 2016	10.5694/mja16.00124
**4**	Codesign to increase implementation of insomnia guidelines in primary care	2022	Kristien Coteur	Elsevier	10.1016/j. pec.2022.08.018
**5**	Knowledge mobilisation: a UK co‐creation study to devise strategies to amend lay and practitioner atopic eczema mindlines to improve consultation experiences and self‐management practices in primary care	2020	Fiona Cowdell	BMJ Open	10.1136/bmjopen‐2019‐036520
**6**	Partnering with patients to improve access to primary care	2019	Sam Davie	BMJ Open	10.1136/bmjoq‐2019‐000777
**7**	Adopting international recommendations to design a model for maternal health service to cope with pandemic disruption for Indonesian primary care	2023	Fitriana Murriya Ekawati	BMC Pregnancy and Childbirth	10.1186/s12884‐023‐05433‐8
**8**	Primary Care Women's Health Screening: A Case Study of a Community Engaged Human Centered Design Approach to Enhancing the Screening Process	2019	Kathleen A. Foley	Maternal and Child Health Journal	10.1007/s10995‐019‐02802‐8
**9**	The use of Coproduction, Codesign and co‐creation to mobilise knowledge in the management of health conditions: a systematic review	2022	Cheryl Grindell	BMC Health Services Research	10.1186/s12913‐022‐08079‐y
**10**	Engaging patients in primary care design: An evaluation of a novel approach to co‐designing care	2019	Erin Hertel	Health Expectations	10.1111/hex.12909
**11**	How Older Persons and Health Care Professionals Co‐designed a Medication Plan Prototype Remotely to Promote Patient Safety: Case Study	2023	Malin Holmqvist	JMIR Aging	10.2196/41950
**12**	Future Health Today: Codesign of an electronic chronic disease quality improvement tool for use in general practice using a service design approach	2020	Barbara Hunter	BMJ Open	10.1136/bmjopen‐2020‐040228
**13**	Empowering people to help speak up about safety in primary care: Using Codesign to involve patients and professionals in developing new interventions for patients with multimorbidity	2018	Sarah Knowles PhD	Health Expectations	10.1111/hex.12648
**14**	Realising radical potential: building community power in primary health care through Participatory Action Research	2023	Denny Mabetha	International Journal for Equity in Health	10.1186/s12939‐023‐01894‐7
**15**	Feasibility, acceptability and initial outcome of implementing community scorecard to monitor community level public health facilities: experience from rural Bangladesh	2020	Shehrin Shaila Mahmood	International Journal for Equity in Health	10.1186/s12939‐020‐01265‐6
**16**	Learning from a Codesign exercise aimed at developing a navigation intervention in the general practice setting	2022	Sabuj K. Mistry	Family Practice	10.1093/fampra/cmac020
**17**	Developing a patient safety guide for primary care: A Codesign approach involving patients, carers and clinicians	2021	Rebecca L. Morris	Health Expectations	10.1111/hex.13143
**18**	Fostering Community Engagement and Acquiring Understanding of Health Needs through a Participatory Rural Appraisal in Haiti	2018	Penelope Neal	John Hopkins University Press	10.1353/cpr.2018.0064
**19**	Community participation for rural healthcare design: description and critique of a method	2016	Amy Nimegeer	University of Glasgow	10.1111/hsc.12196
**20**	Parent‐Professional Co‐development of a Tool to Stimulate Children's Development at Home: The TRIAGE Method	2022	Béatrice Ouellet	Journal of Child and Family Studies	10.1007/s10826‐021‐02030‐1
**21**	Applying participatory design to a pharmacy system intervention	2019	Apoorva Reddy	Elsevier	10.1016/j. sapharm.2018.11.012
**22**	Access, Relationships, Quality and Safety (ARQS): a qualitative study to cocreate an Indigenous patient experience tool for virtual primary care	2023	Pamela Roach	BMJ	10.1136/bmjoq‐2023‐002365
**23**	Recommendations for community pharmacy to improve access to medication advice for people from ethnic minority communities: A qualitative person‐centred Codesign study	2022	Anna Robinson	Health Expectations	10.1111/hex.13611
**24**	Design details for overdose education and take‐home naloxone kits: Codesign with family medicine, emergency department, addictions medicine and community	2022	Kate Sellen	Health Expectations	10.1111/hex.13559
**25**	Co‐designing an intervention to improve the process of deprescribing for older people living with frailty in the United Kingdom	2023	Jonathan Silcock	Health Expectations	10.1111/hex.13669
**26**	Tips and Traps: Lessons from Co‐designing a Clinician E‐Monitoring Tool for Computerised Cognitive Behavioural Therapy	2017	Frederick Sundram	JMIR Mental Health	10.2196/mental.5878
**27**	Using an adaptive, Codesign approach to strengthen clinic‐level immunisation services in Khayelitsha, Western Cape Province, South Africa	2021	Andrea Timothy	BMJ Global Health	10.1136/bmjgh‐2020‐004004
**28**	Persona‐scenario exercise for co‐designing primary care interventions	2014	Ruta Valaitis	Canadian Family Physician	PMCID: PMC3952768
**29**	Health TAPESTRY: co‐designing interprofessional primary care programs for older adults using the persona‐scenario method	2019	Ruta Valaitis	BMC Family Practice	10.1186/s12875‐019‐1013‐9
**30**	Using a Codesign process to develop an integrated model of care for delivering self‐management intervention to multi‐morbid COPD people in rural Nepal	2021	Uday Narayan Yadav	Health Research Policy and Systems	10.1186/s12961‐020‐00664‐z

## 1

PRISMA checklist for scoping reviews.

**Preferred Reporting Items for Systematic reviews and Meta‐Analyses extension for Scoping Reviews (PRISMA‐ScR) Checklist**

**Table:**

SECTION	ITEM	PRISMA‐ScR CHECKLIST ITEM	REPORTED ON PAGE #
TITLE			
Title	1	Identify the report as a scoping review.	Title page
ABSTRACT			
Structured summary	2	Provide a structured summary that includes (as applicable): background, objectives, eligibility criteria, sources of evidence, charting methods, results, and conclusions that relate to the review questions and objectives.	Abstract, page 1.
INTRODUCTION			
Rationale	3	Describe the rationale for the review in the context of what is already known. Explain why the review questions/objectives lend themselves to a scoping review approach.	Introduction, pages 2‐3.
Objectives	4	Provide an explicit statement of the questions and objectives being addressed with reference to their key elements (e.g., population or participants, concepts, and context) or other relevant key elements used to conceptualise the review questions and/or objectives.	Introduction, page 3.
METHODS			
Protocol and registration	5	Indicate whether a review protocol exists; state if and where it can be accessed (e.g., a Web address); and if available, provide registration information, including the registration number.	N/A
Eligibility criteria	6	Specify characteristics of the sources of evidence used as eligibility criteria (e.g., years considered, language, and publication status), and provide a rationale.	Methods, pages 3‐4.
Information sources^a^	7	Describe all information sources in the search (e.g., databases with dates of coverage and contact with authors to identify additional sources), as well as the date the most recent search was executed.	Methods, pages 3‐4.
Search	8	Present the full electronic search strategy for at least 1 database, including any limits used, such that it could be repeated.	Appendix A, page 22.
Selection of sources of evidence^b^	9	State the process for selecting sources of evidence (i.e., screening and eligibility) included in the scoping review.	Abstract, page 1. Methods pages 3‐4.
Data charting process^c^	10	Describe the methods of charting data from the included sources of evidence (e.g., calibrated forms or forms that have been tested by the team before their use, and whether data charting was done independently or in duplicate) and any processes for obtaining and confirming data from investigators.	Methods, pages 3‐4. Results, pages 5‐19.
Data items	11	List and define all variables for which data were sought and any assumptions and simplifications made.	Results, pages 5‐19.
Critical appraisal of individual sources of evidence^d^	12	If done, provide a rationale for conducting a critical appraisal of included sources of evidence; describe the methods used and how this information was used in any data synthesis (if appropriate).	Methods, pages 3‐4.
Synthesis of results	13	Describe the methods of handling and summarising the data that were charted.	Methods, pages 3‐4. Results, pages 5‐19.
RESULTS			
Selection of sources of evidence	14	Give numbers of sources of evidence screened, assessed for eligibility, and included in the review, with reasons for exclusions at each stage, ideally using a flow diagram.	Results, Table 3.
Characteristics of sources of evidence	15	For each source of evidence, present characteristics for which data were charted and provide the citations.	Results, Tables 3, 4, 5.
Critical appraisal within sources of evidence	16	If done, present data on critical appraisal of included sources of evidence (see item 12).	Results, Tables 3, 4, 5.
Results of individual sources of evidence	17	For each included source of evidence, present the relevant data that were charted that relate to the review questions and objectives.	Results, Tables 3, 4, 5.
Synthesis of results	18	Summarise and/or present the charting results as they relate to the review questions and objectives.	Results, Tables 3, 4, 5.
DISCUSSION			
Summary of evidence	19	Summarise the main results (including an overview of concepts, themes, and types of evidence available), link to the review questions and objectives, and consider the relevance to key groups.	Discussion, pages 19‐20.
Limitations	20	Discuss the limitations of the scoping review process.	Limitations, page 20.
Conclusions	21	Provide a general interpretation of the results with respect to the review questions and objectives, as well as potential implications and/or next steps.	Conclusion, page 21.
FUNDING			
Funding	22	Describe sources of funding for the included sources of evidence, as well as sources of funding for the scoping review. Describe the role of the funders of the scoping review.	N/A

Abbreviations: JBI = Joanna Briggs Institute; PRISMA‐ScR = Preferred Reporting Items for Systematic reviews and Meta‐Analyses extension for Scoping Reviews.

*Source:* Tricco AC, Lillie E, Zarin W, O'Brien KK, Colquhoun H, Levac D, et al. PRISMA Extension for Scoping Reviews (PRISMAScR): Checklist and Explanation. Ann Intern Med. 2018;169:467–473. doi: 10.7326/M18‐0850.

^a^ Where *sources of evidence* (see second footnote) are compiled from, such as bibliographic databases, social media platforms, and Web sites.

^b^ A more inclusive/heterogeneous term used to account for the different types of evidence or data sources (e.g., quantitative and/or qualitative research, expert opinion, and policy documents) that may be eligible in a scoping review as opposed to only studies. This is not to be confused with *information sources* (see first footnote).

^c^ The frameworks by Arksey and O'Malley (6) and Levac and colleagues (7) and the JBI guidance (4, 5) refer to the process of data extraction in a scoping review as data charting.

^d^ The process of systematically examining research evidence to assess its validity, results, and relevance before using it to inform a decision. This term is used for items 12 and 19 instead of “risk of bias” (which is more applicable to systematic reviews of interventions) to include and acknowledge the various sources of evidence that may be used in a scoping review (e.g., quantitative and/or qualitative research, expert opinion, and policy document).

## 1

Grey literature list.

1. Agency for Clinical Innovation, NSW Health. Codesign toolkit
2. Agency for Clinical Innovation, NSW Health. 2019. A Guide to Build Codesign Capability
3. Alberta Health Services, Canada. 2020. Understanding Codesign
4. Australia and New Zealand School of Government (ANZSOG). 2024. Nudging citizens and Coproduction
5. Australian Government. 2023. Primary Care Rural Innovative Multidisciplinary Models (PRIMM).
6. Batalden M, Batalden P, Margolis P, Seid M, Armstrong G, Opipari‐Arrigan L, Hartung H. Coproduction of healthcare service. BMJ Qual Saf. 2016 Jul;25(7):509‐17. doi: 10.1136/bmjqs‐2015‐004315. Epub 2015 Sep 16. PMID: 26376674; PMCID: PMC4941163.
7. Buckinghamshire, Oxfordshire and Berkshire West, UK. Transforming Primary Care – Executive Summary
8. Consumers Health Forum of Australia. 2018. Submission: Patient Safety and Quality Improvement in Primary Care
9. Cummings Graduate Institute for Behavioral Health Studies.
10. Elwyn G, Nelson E, Hager A, et al. Coproduction: when users define quality. BMJ Qual Saf 2020;29:711–716.
11. Fusco, F., Marsilio, M. and Guglielmetti, C. Co‐creation in healthcare: framing the outcomes and their determinants. 2023. Journal of Service Management, Vol. 34 No. 6, pp. 1‐26. https://doi.org/10.1108/JOSM-06-2021-0212
12. Goodyear‐Smith F. Use of Codesign in primary care research: real‐life examples. Fam Med Community Health. 2021 Jun 16;9(Suppl 1):e001181. doi: 10.1136/fmch‐2021‐001181. PMCID: PMC8278916.
13. Harris MF et al. Co‐designing a Community Health Navigator program to assist patients to transition from hospital to community. Australian Journal of Primary Health 30. 2024. PY24042. doi:10.1071/PY24042
14. Health Consumers NSW
15. Healthy North Coast. 2021. Re‐imagining Primary Care Access
16. Hunter B, Biezen R, Alexander K, Lumsden N, Hallinan C, Wood A, McMorrow R, Jones J, Nelson C, Manski‐Nankervis JA. Future Health Today: Codesign of an electronic chronic disease quality improvement tool for use in general practice using a service design approach. BMJ Open. 2020 Dec 18;10(12):e040228. doi: 10.1136/bmjopen‐2020‐040228. PMID: 33371024; PMCID: PMC7751202.
17. Institute for Healthcare Improvement
18. Janamian T, Crossland L, Jackson C. Embracing value co‐creation in primary care services research: a framework for success. Med J Aust 2016; 204 (7): S5. doi: 10.5694/mja16.00112.
19. Janamian T, Dawda P, Jammal W. Achieving person‐centred primary health care through value co‐creation. Med J Aust. 2022 Jun 6;216(10):518‐519. doi: 10.5694/mja2.51538. PMID: 35665511.
20. Javanparast, S., Robinson, S., Kitson, A. et al. Embedding research Codesign knowledge and practice: Learnings from researchers in a new research institute in Australia. *Res Involv Engagem* **8**, 71 (2022). https://doi.org/10.1186/s40900-022-00392-4
21. Kealy‐Bateman, W., Gorman, G. M., & Carroll, A. P. (2021). Patient/Consumer Codesign and Coproduction of Medical Curricula: A Possible Path Toward Improved Cultural Competence and Reduced Health Disparity. *SAGE Open*, *11*(2). https://doi.org/10.1177/21582440211016836
22. Kiran T, Ramji N, Derocher MB, Girdhari R, Davie S, Lam‐Antoniades M. Ten tips for advancing a culture of improvement in primary care. BMJ Qual Saf. 2019 Jul;28(7):582‐587. doi: 10.1136/bmjqs‐2018‐008451. Epub 2018 Oct 31. PMID: 30381328; PMCID: PMC6593644.
23. Kjellström S et al. Exploring, measuring and enhancing the Coproduction of health and well‐being at the national, regional and local levels through comparative case studies in Sweden and England: the ‘Samskapa’ research programme protocol. BMJ Open. 2019 Jul 26;9(7):e029723. doi: 10.1136/bmjopen‐2019‐029723. PMID: 31350253; PMCID: PMC6661680.
24. Kuipers SJ, Cramm JM, Nieboer AP. The importance of patient‐centered care and co‐creation of care for satisfaction with care and physical and social well‐being of patients with multi‐morbidity in the primary care setting. BMC Health Serv Res. 2019 Jan 8;19(1):13. doi: 10.1186/s12913‐018‐3818‐y. PMID: 30621688; PMCID: PMC6323728.
25. Mental Health Coordinating Council, Australia. Codesign and Collaboration
26. Middel C et al. Co‐creation in public health research: an introduction to basic principles. 2024. https://doi.org/10.17061/phrp3432419
27. Munce SE et al. Development of the Preferred Components for Codesign in Research Guideline and Checklist: Protocol for a Scoping Review and a Modified Delphi Process. JMIR Res Protoc. 2023 Oct 30;12:e50463. doi: 10.2196/50463. PMID: 37902812; PMCID: PMC10644195.
28. National Health and Medical Research Council, Australia. Partnering Decision Tree
29. National Health Service, UK. Co:Create. 2020. Piloting and developing approaches to Coproduction in Primary Care Networks
30. National Health System Providers, UK. 2024. Coproduction and engagement with communities
31. National Health Service, UK. South Central Ambulance Service. Coproduction and Codesign: A delivery model for the future
32. Network of European Foundations
33. NSW Council of Social Service. 2024. Principles of Codesign
34. NSW Health. 2023. All of Us: A guide to engaging consumers, carers and communities across NSW Health
35. NSW Regional Health Partners. 2024. Doing research together
36. Nuffield Trust, UK
37. Peters, S., Guccione, L., Francis, J. et al. Evaluation of research Codesign in health: a systematic overview of reviews and development of a framework. *Implementation Sci* **19**, 63 (2024). https://doi.org/10.1186/s13012-024-01394-4
38. Robert G, Cornwell J, Locock L, Purushotham A, Sturmey G, Gager M et al. Patients and staff as co‐designers of healthcare services *BMJ* 2015; 350:g7714 doi:10.1136/bmj.g7714
39. Social Care Institute for Excellence, UK. Coproduction
40. The Health Foundation, UK
41. The Health Foundation, UK. 2017. Coproduction: Everybody's talking about it, but am I really doing it?
42. The Health Foundation, UK. What is coproduction?
43. The Kings Fund, UK
44. The Sax Institute, Australia
45. The Transform Initiative
46. Thorburn, K., Waks, S., Aadam, B., Fisher, K. R., Spooner, C., & Harris, M. F. (2024). Creating the conditions for collaborative decision‐making in Codesign. *Codesign*, *20*(4), 567–584. https://doi.org/10.1080/15710882.2024.2349578
47. TransForm: Integrated Community Care
48. TransForm Toolkits. Integrated People‐Centred Health Services
49. Western Victoria Primary Health Network. 2020. After Hours Sub‐regional Codesign
50. World Health Organization. 2018. Quality in primary health care
